# New Anthracenone and Xanthenones From *Tricholaena teneriffae* With Antioxidant Activity: In Vitro and In Silico Studies

**DOI:** 10.1002/cbdv.202503225

**Published:** 2026-01-23

**Authors:** Yosra Belghith, Mayssa Bsaihia, Siwar Soltani, Noureddine Allouche, Imed Mezghani, Hichem Ben Salah

**Affiliations:** ^1^ Faculty of Sciences of Sfax Laboratory of Organic Chemistry LR17ES08 (Natural Substances Team) University of Sfax Sfax Tunisia; ^2^ Environmental Engineering and Ecotechnology Laboratory (LGEET) LR16ES19 National Engineering School of Sfax (ENIS) University of Sfax Sfax Tunisia

**Keywords:** anthracenones, antioxidant activity, molecular docking, *Tricholaena teneriffae*, xanthenones

## Abstract

Three novel phenolic compounds, namely, 6‐hydroxy‐3‐methoxy‐7*H*‐benzo[d]anthracen‐7‐one **(1)**, 3‐hydroxy‐12*H*‐benzo[a] xanthen‐12‐one **(2)**, and 10‐hydroxy‐12*H*‐benzo[a]xanthen‐12‐one **(3)**, have been isolated from the aerial parts of *Tricholaena teneriffae* along with a known benzoxanthenone, 12*H*‐benzo[a]xanthen‐12‐one **(4)**. All structures were elucidated through 1D‐ and 2D‐nuclear magnetic resonance (NMR) and UV analysis. The results revealed that Compounds (**1**–**3**) possess significant antioxidant capacity, slightly weaker but still comparable to that of vitamin C, whereas Compound (**4**), which lacks hydroxyl substituents, exhibited negligible antioxidant activity. Moreover, the antioxidant properties of the isolated compounds were confirmed by in silico docking analysis against lipoxygenase‐3, and it revealed that the isolated compounds effectively bind to the enzyme's active site with favorable binding energies, forming stable hydrogen bonds and hydrophobic interactions. These findings could enhance the understanding of this species rich in natural antioxidants and encourage its potential applications in the development of agricultural and industrial goods.

## Graphical abstract

Three novel phenolic compounds and one known benzoxanthenone are isolated from Tricholaena teneriffae. Compounds (1–3) demonstrate potent antioxidant activity, comparable to that of vitamin C, while compound (4) exhibits negligible activity. Docking studies are employed to validate the binding of the compounds to lipoxygenase‐3, providing evidence of the potential of Tricholaena teneriffae as a source of natural antioxidants for industrial purposes.

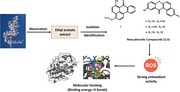

## 1 Introduction

Plants growing in arid and semiarid environments have developed exceptional adaptive mechanisms to tolerate and survive under extreme ecological conditions, including high solar irradiation, saline soil, long‐period water shortage, and low levels of essential nutrients [1, 2]. Desert plants utilize various physiological and biochemical mechanisms to survive under stress conditions, one of which is the biosynthesis of primary and secondary metabolites. Although primary metabolites are essential for basic metabolism and development, secondary metabolites are specialized molecules that increase the adaptive fitness of a plant because they afford protection against abiotic stresses and biotic antagonists (pathogens and herbivores) [3]. They are structurally diverse and functionally versatile acting as natural antibiotics, insecticides, herbicides, antioxidants, and protective agents against external aggressions [4]. On the basis of their biosynthetic origins, secondary metabolites are generally classified into distinct molecular families, including phenolic compounds, alkaloids, terpenes, and steroids [5]. Among them, polyphenolic compounds are of particular importance due to their diverse bioactivities. They are well‐documented for their potent antioxidant properties, their ability to modulate signaling pathways in living organisms, and their wide pharmacological potential [6, 7, 8].

In arid and semiarid habitats, prolonged exposure to drought, thermal stress, and intense solar radiation is known to promote the enhanced biosynthesis of phenolic secondary metabolites, which act as adaptive defense compounds against oxidative and environmental stress. Several studies on xerophytic and desert grasses have reported similar correlations between harsh ecological conditions and the accumulation of polyphenolic structures with antioxidant functions, supporting the ecological relevance of the phenolic profile observed in *Tricholaena teneriffae* [2, 4, 5].

Anthracenones and xanthenones are two structurally related classes of polycyclic phenolic compounds that occur mainly in a limited number of plant families, such as Rubiaceae, Clusiaceae, Hypericaceae, Rhamnaceae, and Gentianaceae [9, 10, 11]. These metabolites exhibit broad pharmacological activities, including antioxidant, anti‐inflammatory, antimicrobial, and cytotoxic effects, largely attributed to their conjugated aromatic frameworks and phenolic hydroxyl groups [12, 13, 14]. Owing to their structural diversity and bioactivity, they are often considered chemotaxonomic markers and valuable structures in drug discovery.

Despite this chemical interest, anthracenone‐ and xanthenone‐type metabolites remain extremely uncommon in Poaceae family. Only a few isolated xanthenone derivatives have been reported from *Cymbopogon* and *Phyllostachy*s spp. [15, 16], and no anthracenones have been documented so far in this family. In this regard, *T. teneriffae*, a Saharan wild grass species belonging to the Poaceae family, represents an interesting candidate for phytochemical exploration. It is a species characterized by large ecological amplitudes allowing it to survive in severe environmental conditions. This study represents an important continuation of previous research, which has already established the antibacterial and antioxidant properties of *T. teneriffae*, through LC–ESI–MS profiling, the presence of eight phenolic acids and 13 flavonoids was revealed, indicating a diverse profile of secondary metabolites [1].

**FIGURE 1:**
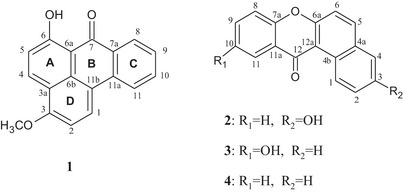
Chemical structures of Compounds **1**–**4** isolated from *Tricholaena teneriffae*.

To better develop this endemic Tunisian herb, this study aimed to describe the isolation and structural elucidation of four compounds from the aerial parts of *T. teneriffae*, which includes three previously undescribed phenolic Compounds (**1–3**) and a known xanthenone derivative (**4**) (Figure 1). All compounds were also assessed for antioxidant capacity in order to correlate the potential bioactivity with their structural features. Additionally, molecular docking models were also conducted to investigate structure–activity relationships (SAR) among the compounds in order to mechanistically suggest potential interactions between the metabolites and biological targets.

## 2 Results and Discussion

### 2.1 Structural Identification of the Isolated Compounds

The strategy for isolation developed a successive maceration of dried aerial parts with solvents increasingly polar in nature (*n*‐hexane, ethyl acetate [EtOAc], and methanol). Choosing an EtOAc extract weighing 2.23 g was based on its intermediate polarity which was helpful in obtaining polyphenolic compounds such as flavonoids and polycyclic aromatics as well as for identifying and isolating other less polar phenolic compounds during chromatography. Column chromatography was performed on silica gel in a stepwise manner using *n*‐hexane‐EtOAc and EtOAc–MeOH gradient elution. Chromatographic fractionation of the EtOAc extract of *T. teneriffae* led to the isolation and structural characterization of three novel phenolic compounds: 6‐hydroxy‐3‐methoxy‐7*H*‐benzo[d]anthracen‐7‐one (**1**), 3‐hydroxy‐12*H*‐benzo[a]xanthen‐12‐one (**2**), and 10‐hydroxy‐12*H*‐benzo[a]xanthen‐12‐one (**3**). Additionally, a previously reported xanthenone identified as 12*H*‐benzo[a] xanthen‐12‐one (**4**) was isolated and characterized for the first time in its natural occurring form.

Compound (**1**) was obtained as a dark yellow amorphous powder. HRESIMS spectrum (Figure S8, Supplementary material) exhibited a pseudo‐molecular ion peak [M+H]⁺ at m/z 277.0478, consistent with the molecular formula C_18_H_12_O_3_. Its molecular formula was determined as C_18_H_12_O_3_ indicating thirteen degrees of unsaturation. The UV spectrum of Compound (**1**) in methanol (Figure S1) shows five absorption maxima at 225, 265, 303, 331, and 383 nm indicating the presence of aromatic groups.

The ^1^H nuclear magnetic resonance (NMR) spectrum (Figure S2) showed a methoxyl group (CH_3_O) at *δ*_H_ 3.92 (3H, s), a phenolic hydroxyl (OH) group at *δ*_H_ 9.92 (1H, s), and four doublets at *δ*_H_ 9.61 (H, d, ^3^ *J* = 9.3 Hz, H‐5), 8.46 (H, d, ^3^ *J* = 9.4 Hz, H‐2), 7.72 (H, d, ^3^ *J* = 9.4 Hz, H‐1), and 7.46 (H, d, ^3^ *J* = 9.3 Hz, H‐4). A split doublet observed at *δ*_H_ 7.54 (1H, ddd, ^3^ *J* = 7.8 and 7.2 Hz; ^4^ *J* = 1 Hz, H‐9) was attributed to an aromatic proton that correlates with two neighboring protons in *ortho* position and with a third proton via a *meta* coupling. In addition, a split doublet at *δ*_H_ 8.29 (1H, dd, ^3^ *J* = 7.8 Hz, ^4^ *J* = 1.4 Hz, H‐8) corresponds to an aromatic proton that correlates with two protons in *ortho* and *meta* positions.

**FIGURE 2:**
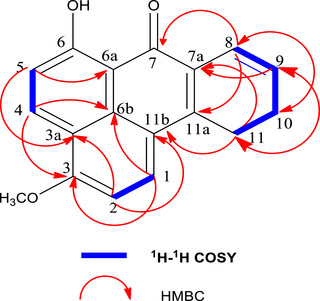
Key heteronuclear multiple bond coherence (HMBC) and ^1^H‐^1^H correlated spectroscopy (COSY) correlations of Compound **(** **1)**.

Combined analysis of the ^1^ ^3^C NMR, DEPT, and HSQC spectra (Figures S3–S5) revealed a total number of 18 carbon signals, including a carbonyl carbon at *δ*_C_ 177.8, one signal for the methoxy group (*δ*_C_ 61.2, CH_3_O), eight aromatic methine groups (*δ*_C_ 118.3, 118.7, 122.4, 122.8, 125.0, 126.4, 130.3, and 135.0), and nine quaternary carbons (*δ*_C_ 114.3, 123.2, 124.8, 126.4, 141.3, 147.1, 154.6, 156.2, and 177.8) (Table 1). Detailed analysis of the NMR data revealed that Compound **(** **1)** belongs to the family of benzanthrones. In heteronuclear multiple bond coherence (HMBC) spectrum (Figure S6), the position of the methoxy group was determined by observing a correlation spot between the methoxy protons at *δ*_H_ 3.92 and the carbon at *δ*_C_ 141.3 (C‐3). The additional HMBC correlations provided crucial information for determining the connectivity of ring A with the rest of the polycyclic system. In ring D, the aromatic proton H‐4 (*δ*_H_ 7.46) demonstrated long‐distance interactions with two quaternary carbon signals at *δ*_C_ 124.8 (C‐6b) and *δ*_C_ 141.3 (C‐3). Additionally, H‐5 (*δ*_H_ 9.61) exhibited distinct HMBC interactions (Figure 2) with two olefinic carbons at *δ*_C_ 114.3 (C‐3a) and *δ*_C_ 147.1 (C‐6a), confirming the fusion of ring A with rings B and D at positions C‐3a, C‐6a, and C‐6b. Furthermore, the proton H‐8 (*δ*_H_ 8.29) has cross peaks in the HMBC spectrum with carbons C‐10 (*δ*_C_ 135.0), C‐11a (*δ*_C_ 154.6), and C‐7 (*δ*_C_177.8). In fact, the proton H‐9 (*δ*_H_ 8.29) exhibited two correlations with C‐11 (*δ*_C_ 118.3) and C‐7a (*δ*_C_ 123.2).

**TABLE 1:** ^1^H (400 MHz) and ^13^C (100 MHz) nuclear magnetic resonance (NMR) data of Compound (**1**).

Position	1	
	*δ*_C_ (ppm)	*δ*_H_ (ppm) (m, *J* in Hz)
1	130.3	8.46 (d, 1H, 9.4)
2	118.7	7.72 (d, 1H, 9.4)
3	141.3	—
3a	114.3	—
4	122.4	7.46 (d, 1H, 9.3)
5	122.8	9.61 (d, 1H, 9.3)
6	126.4	—
6a	147.1	—
6b	124.8	—
7 (CO)	177.8	—
7a	123.2	—
8	126.4	8.29 (dd, 1H, 7.8, 1.4)
9	125.0	7.54 (ddd, 1H, 7.8, 6.5, 1.0)
10	135.0	7.89 (td, 1H, 6.5, 1.4)
11	118.3	7.75 (dd, 1H, 6.5, 1.0)
11a	154.6	—
11b	156.2	—
(OH)	—	9.92 (s, 1H)
OCH3	61.2	3.92 (s, 3H)

Moreover, in the ^1^H‐^1^H correlated spectroscopy (COSY) spectrum (Figure S7), three significant spin coupling systems (H1/H2), (H4/H5), and (H8/H9, H9/H10, and H10/H11) were unambiguously observed. These combined data unequivocally established the tetracyclic framework of Compound (**1**), which was structurally identified as 6‐hydroxy‐3‐methoxybenzo[d]anthracen‐7‐one, a novel anthracenone. This compound was classified as a benzanthrone based on its tetracyclic aromatic core and the presence of a characteristic carbonyl group at C‐7, which defines benzanthrone structures. For consistency, the term benzanthrone is used throughout the manuscript.

These assignments were confirmed by key diagnostic COSY and HMBC correlations, which were decisive in distinguishing the new structures from closely related xanthenone and anthracenone analogues. For Compound **(** **1)**, the H‐4/H‐5 and H‐8/H‐9/H‐10 spin systems observed in the COSY spectrum, together with the HMBC cross‐peaks from H‐5 to C‐3a and C‐6a and from the methoxy protons to C‐3, unambiguously established the benzanthrone core and the position of the methoxyl substituent. Likewise, for Compounds **(** **2)** and **(** **3)**, the hydroxyl substitution patterns were confirmed by the HMBC correlations from H‐1 and H‐4 to C‐3 (Compound **2**) and from H‐9 and H‐11 to C‐10 (Compound **3**), which clearly differentiated these molecules from the parent xanthenone scaffold and from reported structural isomers.

Compounds (**2–4**) were yellow amorphous powders. Compound (**4**) was identified as 12*H*‐benzo[a]xanthen‐12‐one through a comparison of its spectroscopic data with those reported in the literature [17, 18]. It is worth mentioning that Compound (**4**) was isolated for the first time in its natural occurring form.

By comparing the NMR data of Compounds (**2**) and (**3**) (Table 2) with those of 12*H*‐benzo[a] xanthen‐12‐one (**4**), it was determined that these two compounds had an additional hydroxyl group at C‐3 and C‐10, respectively. The presence of these hydroxyl groups at C‐3 and C‐10 was confirmed through their HMBC spectra (Figures S12 and S18). This was supported by the correlations between protons H‐1 (*δ*_H_ 9.83) and H‐4 (*δ*_H_ 7.36) to C‐3 (*δ*_C_ 156.0) for Compound (**2**), and between protons H‐9 (*δ*_H_ 7.35) and H‐11 (*δ*_H_ 7.60) to C‐10 (*δ*_C_ 148.3) for Compound (**3**). Therefore, Compounds (**2**) and (**3**) were identified as 3‐hydroxy‐12*H*‐benzo[a]xanthen‐12‐one and 10‐hydroxy‐12*H*‐benzo[a]xanthen‐12‐one molecules, respectively. After conducting a comprehensive bibliographic review, it has been confirmed that these two Compounds (**2–3**) have not been previously identified.

**TABLE 2:** ^1^H (400 MHz) and ^13^C (100 MHz) nuclear magnetic resonance (NMR) data of Compounds **(** **2**–**4**).

Position	2		3		4	
	*δ*_C_ (ppm)	*δ*_H_ (ppm) (m, *J* in Hz)	*δ*_C_ (ppm)	*δ*_H_ (ppm) (m, *J* in Hz)	*δ*_C_ (ppm)	*δ*_H_ (ppm) (m, *J* in Hz)
1	128.3	9.83 (d, 1H, 9.2)	126.7	10.0 (d, 1H, 8.5)	127.0	10.14 (d, 1H, 8.5)
2	121.2	7.38 (m, 1H)	130.1	7.82 (t, 1H, 8.5)	129.6	7.82 (td, 1H, 8.5, 1.5)
3	156.1	—	126.4	7.68 (t, 1H, 8.5)	128.4	7.63 (t, 1H, 8.5)
4	111.5	7.36 (m, 1H)	129.3	8.11 (d, 1H, 8.5)	130.1	7.95 (d, 1H, 8.5)
4a	132.6	—	130.5	—	130.5	—
4b	125.2	—	130.7	—	131.1	—
5	136.8	8.23 (d, 1H, 9.1)	137.3	8.39 (d, 1H, 9.0)	136.7	8.18 (d, 1H, 8.8)
6	119.0	7.70 (d, 1H, 9.1)	119.0	7.75 (d, 1H, 9.0)	118.0	7.63 (d, 1H, 8.8)
6a	156.0	—	157.7	—	157.7	—
7a	154.8	—	155.0	—	154.7	—
8	118.7	7.74 (d, 1H, 8.0)	119.7	7.65 (d, 1H, 9.0)	117.5	7.61 (d, 1H, 7.7)
9	135.0	7.89 (t, 1H, 8.0)	124.3	7.34 (dd, 1H, 9.0, 2.8)	134.0	7.77 (t, 1H, 7.7)
10	125.2	7.54 (t, 1H, 8.0)	148.4	—	124.3	7.49 (t, 1H, 7.7)
11	126.4	8.30 (d, 1H, 8.0)	109.1	7.60 (d, 1H, 2.8)	126.7	8.47 (d, 1H, 7.7)
11a	123.1	—	124.1	—	123.6	—
12 (CO)	178.1	—	177.8	—	178.6	—
12a	114.4	—	113.2	—	114.7	—
(OH)	—	10.00 (s)	—	10.00 (s)	—	—

This discovery greatly enhances the phytochemical profile of the genus. In contrast to typical phenolic acids and flavonoids found in *T. teneriffae* and other members of the Poaceae family, anthracenone‐ and xanthenone‐type scaffolds are very uncommon within this taxonomic context. The occurrence of 12*H*‐benzo[a]xanthen‐12‐one (**4**) in combination with the other three new Compounds (**1–3**) further emphasizes the high degree of chemical diversity exhibited by the species, indicating that *T. teneriffae* has an extraordinarily diverse range of polycyclic phenolic compounds. As phenolic compounds are highly relevant secondary metabolites known for their diverse bioactivities, including potent antioxidant properties, these findings provide a crucial basis for future attempts at establishing SARs, as well as the basis for developing new applications using these novel and biologically active natural scaffolds [19].

### 2.2 Antioxidant Assays

The isolated compounds were subjected to rigorous evaluation for antioxidant activity using 2,2‐diphenyl‐1‐picrylhydrazyl (DPPH) radical scavenging, ferric‐reducing antioxidant power (FRAP), and total antioxidant capacity (TAC) in vitro methods. DPPH measures electron‐ or hydrogen‐donating capacity, TAC assesses overall reducing power (vitamin E equivalents), and FRAP quantifies ferric‐reducing potential. The combination of these methods provides comprehensive view of antioxidant effect as each differs in redox and free radical reaction mechanisms of redox and free radical reactions.

The results of the DPPH test showed that Compounds **1**, **2**, and **3** exhibited strong radical scavenging activity, with respective IC_50_ values of 145 ± 70, 145 ± 19, and 137 ± 27 µM, respectively (Table 3). The antioxidant activity of Compounds **(** **1–3)** is similar but slightly weaker than that of vitamin C (IC_50_ –= 125 ± 57 µM). These compounds denote their strong capacity to donate electrons and efficiently scavenge DPPH radicals. The results of the TAC assay also aligned with these findings. Compounds **1**, **2**, and **3** had high antioxidant activities calculated to be 624.85, 540.44, and 607.15 mg of vitamin E equivalents per gram of sample (Table 3). These values reveal radical scavenging activity at levels similar to polyphenolic compounds and other well‐known antioxidants. Likewise, the FRAP assay showed similar results (Table 3). Decreasing reducing activity, expressed as EC_50_, occurred in the following order: vitamin C (590 ± 11 µM) > Compound **(** **1)** (2200 ± 15 µM) > Compound **(** **3)** (2503 ± 27 µM) > Compound **(** **2)** (3143 ± 23 µM). The close agreement among the three independent assays provides strong evidence that Compounds (**1–3**) are the principal contributors to the antioxidant activity of *T. teneriffae*. Compound (**4**) exhibited only a very weak activity by the TAC test, insufficient to reach 50% scavenging or reducing capacity in DPPH and FRAP assays. Therefore, it is considered practically inactive.

**TABLE 3:** Antioxidant activity of isolated Compounds **(** **1**–**4)**.

Compound	DPPH IC_50_ (µM)	FRAP EC_50_ (µM)	Total antioxidant capacity^a^
**1**	145 ± 70	2200 ± 15	624.85 ± 0.01
**2**	145 ± 19	3143 ± 23	540.44 ± 0.02
**3**	137 ± 27	2503 ± 27	607.15 ± 0.08
**4**	Inactive	Inactive	17.09 ± 0.05
**Vitamin C**	125 ± 57	590 ± 11	—

*Note*: Values expressed are means ± SD of three parallel measurements (*p *< 0.05).

Abbreviations: DPPH, 2,2‐diphenyl‐1‐picrylhydrazyl; FRAP, ferric‐reducing antioxidant power.

^a^mg vitamin E/g of compounds.

When compared to the antioxidant values reported for representative phenolic natural products, the activities of Compounds (**1–3**) fall within the range of moderately to strongly active plant‐derived antioxidants. For example, the reported IC_50_ values for common flavonoids and phenolic acids typically range from 120 to 300 µM in the DPPH assay [20] and 2000–4000 µM in FRAP measurements [21], depending on structural features such as hydroxyl substitution and conjugation patterns. In this context, the DPPH and FRAP responses of Compounds (**1–3**) are comparable to, and in some cases stronger than, several well‐documented phenolic antioxidants isolated from medicinal plants [22, 23]. However, they are slightly weaker than vitamin C, which is generally regarded as a highly potent reference antioxidant in both assays [24]. Overall, these comparisons suggest that the antioxidant responses of Compounds **(** **1–3** **)** can be classified as moderate to strong relative to recognized natural antioxidant benchmarks. Furthermore, their activity levels can be considered biologically meaningful in systems where phenolic‐based antioxidant protection is required.

The activity of phenolic Compounds (**1–3**) can be directly linked to certain features of their structures, particularly the presence of hydroxyl substituents. These groups are well known in the biochemical literature for their critical antioxidant functions, particularly in stabilizing reactive oxygen species (ROS) and other free radicals through the electron delocalization phenomenon in certain aromatic systems. In fact, the fundamental electronic stabilization of free radicals leads to two core mechanistic processes: (i) hydrogen atom transfer (HAT), in which a hydroxyl group quenches a radical by “donating” a hydrogen atom to generate a resonance‐stabilized phenoxy radical, and (ii) single electron transfer (SET), in which the hydroxyl group donates an electron, neutralizing the free radical [19, 25]. In contrast, the antioxidant differences among Compounds (**1–3**) arise from the position of their OH groups. Compound (**1**) carries a single hydroxyl at C‐8, located *ortho* to the carbonyl, which favors electron transfer and explains its relatively better FRAP value. Compound (**2**) has one OH at C‐11, but its position does not allow strong resonance stabilization, resulting in weaker radical‐scavenging efficiency. Compound (**3**), the most active in DPPH, possesses a hydroxyl at C‐1, positioned in an *ortho*/*para*‐arrangement relative to the oxygen bridge and aromatic system, which enhances delocalization of the resulting phenoxyl radical and promotes efficient HAT. These structural distinctions account for the observed variations in IC_50_/EC_50_ values among Compounds **(1–3)**. Although all three compounds possess a single –OH group capable of participating in HAT and SET mechanisms, their efficiency depends on the degree of resonance stabilization of the resulting phenoxyl radical. In Compound (**1**), the hydroxyl group is located *ortho* to the conjugated carbonyl, favoring electron delocalization and explaining its stronger reducing capacity. In Compound (**2**), the hydroxyl group is positioned in a less conjugated environment, which limits radical stabilization and results in lower activity. In contrast, Compound **(** **3** **)** benefits from a more extended conjugation pathway, allowing greater stabilization of the phenoxyl intermediate and leading to its comparatively higher DPPH scavenging capacity.

Beyond the positional effect of the hydroxyl group, the antioxidant behavior of Compounds (**1–3**) can also be interpreted in terms of qualitative electronic effects. The most active structures display a higher degree of π‐conjugation and planarity, which facilitates delocalization of the resulting phenoxyl radical across the aromatic system. This extended resonance stabilization lowers the energetic cost of hydrogen atom or electron donation, thereby enhancing both HAT and SET reactivity. Conversely, when the hydroxyl group is located in a position with weaker conjugation or reduced coplanarity, charge delocalization is restricted and the radical intermediate is less stabilized, which is consistent with the lower antioxidant response observed. The lack of activity of Compound (**4**), which contains no hydroxyl substituents, confirms that it cannot engage in either HAT or SET mechanisms.

These anthracenone and xanthenone structures are much less common than the phenolic acid and flavonoid structures routinely found in both *T. teneriffae* and all other species of Poaceae. The occurrence of 12*H*‐benzo[a]xanthen‐12‐one (4) further increases the species’ chemical diversity, highlighting its unusually rich polycyclic phenolic profile. Antioxidant levels have an important impact on the applied chemistry. The potency comparable to vitamin C, coupled with the structural novelty of the anthracenone and xanthenones, makes them highly promising natural antioxidants. Thus, this significant antioxidant effect from these products will provide ample opportunity for their potential use as ingredients in agricultural and industrial products where they will be required for stable and effective antioxidant preservation.

### 2.3 Molecular Docking Study

In order to predict the antioxidant activity of the isolated compounds, a molecular docking study was performed. Lipoxygenase‐3 (LOX‐3) was selected as a target for evaluating the antioxidant activity of the tested compounds due to its central role in catalyzing the oxidation of polyunsaturated fatty acids, a primary source of ROS and lipid peroxides in biological systems. Inhibition of LOX‐3 can therefore directly reduce the formation of free radicals and oxidative products, providing a mechanistically relevant measure of antioxidant potential. Moreover, LOX‐3 has a well‐characterized catalytic site, making it suitable for molecular docking studies. Its involvement in oxidative‐stress‐related processes, such as inflammation and cellular aging, further highlights the biological significance of this target [26].

**FIGURE 3:**
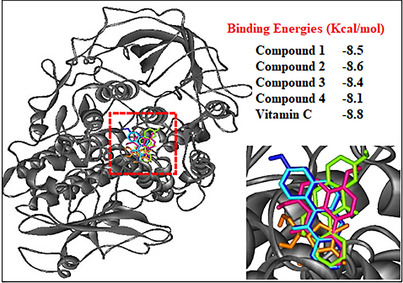
Docking simulation of the binding position of Compound **1** (Pink), Compound **2** (blue), Compound **3** (green), Compound **4** (cyan), and vitamin C (orange) in the active site of lipoxygenase‐3 protein target with their respective binding energies.

To gain deeper insight into the binding behavior of the tested compounds, a superposition of their docked conformations within the active site of the target protein was assessed. As illustrated in Figure 3, all four compounds occupy the same binding pocket but exhibit slight differences in orientation and spatial arrangement. These differences stem from variations in their aromatic substituents and functional groups, which significantly influence their interaction profiles. As shown in Figure 3, the four compounds exhibited favorable binding energies, ranged from −8.8 to −8.1 kcal/mol. Among them, vitamin C displayed the highest binding stability (−8.8 kcal/mol), followed closely by Compounds (**2**) (−8.6 kcal/mol), then (**1**) (−8.5 kcal/mol) and (**3**) (−8.4 kcal/mol), which reflects their potential affinity for the active site of the target protein.

**FIGURE 4:**
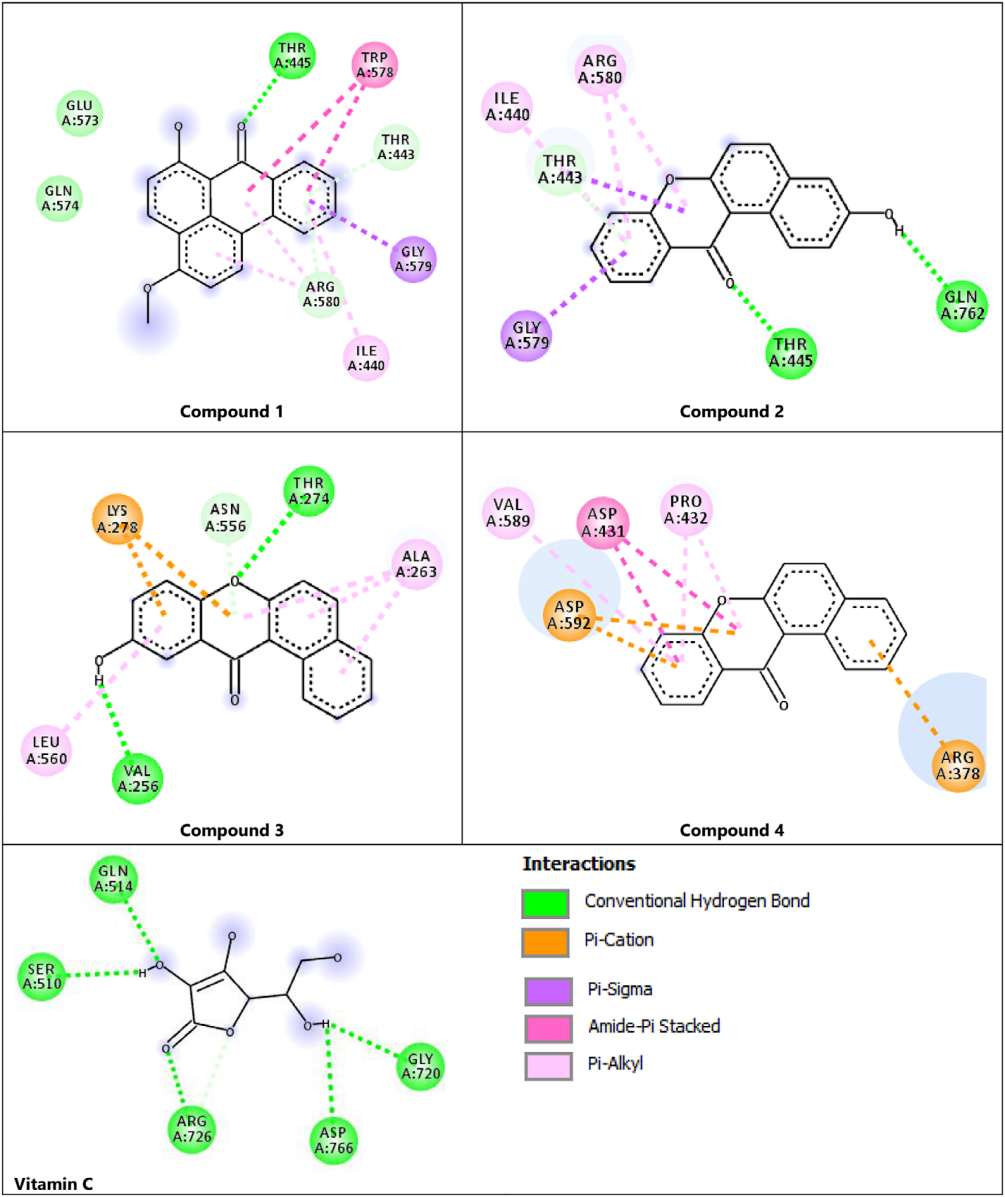
Binding energies and 2D representations of the interaction between vitamin C and the isolated compounds of *Tricholaena teneriffae* with the active site of the lipoxygenase‐3 target.

The detailed 2D representations shown in Figure 4 further elucidate these findings. Compound (**2**) forms a particularly stable complex with the protein, due to its various polar and hydrophobic interactions. It forms two hydrogen bonds with GLN 762 and THR 445, alongside five π‐interactions with ILE 440 and GLY 579 and ARG 580. Similarly, this compound formed stabilizing hydrogen bond with THR A:445 and multiple hydrophobic interactions such as π–alkyl and amide–π stacking with TRP 578, ILE 440, GLY 579, and ARG 580. Compound (**3**) engaged in two hydrogen bonds with the residues THR 274 and ASN 556 and significant π–cation interactions with LYS 278 and four hydrophobic interactions with ALA 263 and LEU 560, indicating a favorable electrostatic contribution to binding stability. Lastly, Compound (**4**) showed a binding profile dominated by electrostatic, notably with ARG A:378 and ASP A:592, and hydrophobic interactions with ASP 431, PRO 432, and VAL 589. Although Compound **(** **4** **)** shows favorable docking scores, it exhibits negligible antioxidant activity in vitro. This discrepancy underscores that molecular docking provides only a static prediction of binding affinity to the target protein and does not capture the full complexity of experimental activity. In vitro antioxidant assays depend not only on target binding but also on kinetic factors, compound solubility, stability, and cell or assay accessibility [27, 28]. Moreover, specific chemical features, such as the presence and position of hydroxyl (–OH) groups, are essential for HAT or SET mechanisms underlying radical scavenging. Without these features, a compound may bind to the enzyme but fail to participate in the chemical reactions required for effective antioxidant activity.

It is therefore important to note that the docking simulations are interpreted as complementary structural evidence and do not directly model redox‐based antioxidant mechanisms such as HAT or SET; consequently, the docking results are considered supportive and must be evaluated in parallel with the in vitro antioxidant assays.

Accordingly, the docking results are interpreted as complementary structural support to the chemical assays. A limitation of the current study is that the antioxidant activity was tested only through complementary chemical methods in vitro (DPPH, FRAP, and TAC). To improve the biological relevance of these compounds, additional testing would need to be conducted, including cellular ROS scavenging studies, detailed enzymatic inhibition kinetics (i.e., determining IC_50_ for LOX‐3), and preliminary cytotoxicity tests to understand their safety profile.

## 3 Conclusions

In summary, this investigation successfully led to the isolation and structural characterization of three new phenolic compounds, including one anthracenone (**1**) and two xanthenones (**2** and **3**), along with a known benzoxanthenone (**4**) from *T. teneriffae's* aerial parts. Only Compounds (**1–3**) exhibited excellent antioxidant capacity in vitro when compared to the positive control (vitamin C). Their antioxidant mechanism was supported through in silico molecular docking study against LOX‐3, which revealed favorable binding energies and stable interactions, particularly for Compound (**2**), highlighting its potential antioxidant action. Collectively, these findings support their potential applications in industries requiring protection against oxidative damage, including cosmetics, pharmaceuticals, and agricultural or industrial products. The discovery of these novel anthracenone and xanthenone scaffolds is significant not only for their potent biological activity but also for their chemotaxonomic relevance. The SAR analysis indicates that antioxidant activity is mainly determined by the positional and electronic context of the hydroxyl group. Future research should aim to translate this strong in vitro chemical activity into biological relevance by assessing cellular ROS assays, LOX‐3 inhibition kinetics, and using realistic cytoprotective and cytotoxicity models in cell cultures.

## 4 Materials and Methods

### 4.1 Plant Material

The aerial part of *T. teneriffae* was collected in late June 2023 from the region of the Bou‐Hedma National Park (Sidi Bouzid [33°30′ N and 9°38′ E], South of Tunisia). The plant material was taxonomically identified by Prof. Mohamed Chaieb, Professor in the Department of Botany, Faculty of Sciences of Sfax. A voucher specimen (LCSN 133) was deposited at the Herbarium of the Organic Chemistry Laboratory (Natural Products Unit), Faculty of Sciences of Sfax, University of Sfax, Tunisia. The harvested plant was dried and stored in closed vials protected from light and heat until analyses.

### 4.2 Extraction and Isolation

The air‐dried plant material (320 g of aerial parts) was successively macerated three times until exhaustion with 200 mL of *n*‐hexane, EtOAc, and methanol for 24 h at room temperature. The extracts were then concentrated under reduced pressure at 40°C to yield dried *n*‐hexane (2.87 g, 0.896% yield), EtOAc (2.23 g, 0.696% yield), and methanol (16.65 g, 5.203% yield) extracts. The crude EtOAc extract was separated using chromatography on a silica gel column (140 g of stationary phase) and eluted with increasing proportions of *n*‐hexane‐AcOEt (100:0; 90:10; 85:15; 80:20; 70:30; 60:40; 50:50; 30:70; 10:90; 0:100) followed by higher polarities using EtOAc–MeOH (99:1; 98:2; 90:10; 80:20) to yield 38 fractions (*F*1–38). Fraction *F*22 (68 mg) exhibited precipitation. Successive solid–liquid washing with solvents of different polarity (*n*‐hexane 100%, *n*‐hexane‐EtOAc 98:2, and *n*‐hexane‐DCM 98:2) produced a subfraction that separated as a yellow, solid powder (45 mg). This subfraction was purified using preparative TLC plates, yielding four pure Compounds: **1** (10 mg, yellow powder, 0.0031% yield), **2** (6 mg, light yellow powder, 0.0018% yield), **3** (3 mg, yellow powder, 0.0009% yield), and **4** (8 mg, yellow powder, 0.0030% yield).

**6‐hydroxy‐3‐methoxybenzo[d]anthracen‐7‐one** (**1**): Yellow amorphous powder, HR‐ESI‐MS: *m/z* 277.0478 [M + H]^+^ (calcd. for C_18_H_13_O_3_, 277.0864); UV (CHCl_3_) *λ*_max_: 225, 265, 303, 331, and 383 nm; ^1^H NMR (DMSO‐d6, 400 MHz) and ^13^C NMR (DMSO‐d6, 100 MHz) data see Table 1.

**3‐hydroxy‐12*H*‐benzo[a]xanthen‐12‐one** (**2**): Yellow powder, HR‐ESI‐MS: *m/z* 263.0729 [M + H]^+^ (calcd. for C_17_H_11_O_3_, 263.0708); UV (CHCl_3_) *λ*_max_: 218, 232, 262, 303, 323, and 382 nm; ^1^H NMR (DMSO‐d6, 400 MHz) and ^13^C NMR (DMSO‐d6, 100 MHz) data see Table 2.

**10‐hydroxy‐12*H*‐benzo[a]xanthen‐12‐one** (**3**): Yellow powder, HR‐ESI‐MS: *m/z* 263.0729 [M + H]^+^ (calcd. for C_17_H_11_O_3_, 263.0708); UV (CHCl_3_) *λ*_max_: 214, 238, 267, 275, 303, and 335 nm; ^1^H NMR (DMSO‐d6, 400 MHz) and ^13^C NMR (DMSO‐d6, 100 MHz) data see Table 2.

**12*H*‐benzo[a] xanthen‐12‐one** (**4**): Yellow powder, HR‐ESI‐MS: *m/z* 247.9815 [M + H]^+^ (calcd. for C_17_H_11_O_2_, 247.0759); ^1^H NMR (CDCl_3_, 400 MHz) and ^13^C NMR (CDCl_3_, 100 MHz) data see Table 2.

### 4.3 Spectroscopic Measurements

#### 4.3.1 Nuclear Magnetic Resonance Spectroscopy Analysis

1D and 2D NMR spectra of isolated compounds were acquired using a Bruker Ascend 400 MHz spectrometer. ^1^H and ^13^C NMR spectra were recorded at the two spectrometer frequencies 400 and 100 MHz, respectively. The data analysis was conducted using MestReNova 5.3.0 software (Mestrelab Research S.L.). Chemical shifts (*δ*) corresponding to the location of the signal in the NMR spectrum are expressed in ppm and coupling constants (*J*) are given in Hz.

#### 4.3.2 Mass Spectrometry Analysis

Helios Omega ThermoScientific spectrophotometer was used to record steady‐state absorption spectra and Perkin Elmer LS55 spectrofluorometer to measure steady‐state emission spectra. All spectroscopic measurements were performed in a rectangular quartz cell measuring 10 mm × 10 mm with a capacity of 3.5 mL, with the emission spectrum being recorded at the maximum absorption (slit width: 1 nm for both emission and excitation).

#### 4.3.3 UV/Visible Spectroscopy Analysis

Methanol was used as the solvent in determining the UV absorption spectra on a spectrophotometer, Varian Cary 100, where each absorption is reported to its corresponding wavelength (nm).

### 4.4 Antioxidant Activities

The antioxidant potential of the isolated compounds was evaluated using three complementary in vitro assays: FRAP, DPPH radical scavenging activity, and TAC. All measurements were performed in triplicate. Vitamin C was used as the sole reference standard for the DPPH and FRAP assays. For these two assays, the results were expressed as 50% inhibitory concentration (IC_50_) and effective concentration (EC_50_), respectively, and the values were calculated from concentration–response curves using nonlinear regression. TAC results were expressed as mg vitamin E equivalents per gram of compound (mg VEE/g).

#### 4.4.1 Ferric‐Reducing Antioxidant Power

The ferric‐reducing capacity of the tested compounds was determined according to the method described by Chelly et al. [29], with slight modifications. Briefly, 1 mg of each compound was dissolved in 1 mL of ethanol and mixed with 2.5 mL of 0.2 M phosphate buffer (pH 6.6) and 2.5 mL of 1% (w/v) potassium ferricyanide solution. The mixture was incubated at 50°C for 20 min, followed by centrifugation for 10 min. A 2.5 mL aliquot of the supernatant was then combined with 2.5 mL of distilled water and 0.5 mL of 0.1% (w/v) ferric chloride solution. The absorbance of the resulting solution was measured spectrophotometrically at 700 nm.

#### 4.4.2 DPPH Radical Scavenging Activity

Free radical scavenging activity was assessed using the DPPH assay, following the protocol of Felhi et al. [30]. For each compound stock solutions (1 mg/mL in methanol) were prepared and serially diluted (0.005–1.000 mg/mL). Then, 1 mL of each concentration sample was mixed with 2 mL of a 0.1 mM methanolic solution of DPPH. The mixtures were incubated at room temperature in the dark for 30 min, after which absorbance was measured at 517 nm. Vitamin C was used as positive controls. Radical scavenging activity was expressed as the percentage inhibition of DPPH radicals using the following formula:

$$\mathrm{Inhibition}\;\left(\%\right)=\frac{A_{\mathrm{control}}-A_{\mathrm{sample}}}{A_{\mathrm{control}}}\;\times 100$$

where $A_{\mathrm{control}}$ represents the absorbance of the control (methanol solution without sample), $A_{\mathrm{sample}}$ represents the absorbance of the test solution.

#### 4.4.3 Total Antioxidant Capacity

The TAC of the isolates was evaluated following the method of Mezghani et al. [1]. A volume of 0.1 mL of each compound solution (1 mg/mL) was added to 1 mL of reagent mixture containing 28 mM sodium phosphate, 0.6 M sulfuric acid, and 4 mM ammonium molybdate. The reaction mixtures were incubated in a boiling water bath at 95°C for 90 min. After cooling to room temperature, the absorbance was measured at 695 nm. The antioxidant capacity was expressed as mg vitamin E equivalents per gram of compound (mg GAE/g of compound).

### 4.5 Molecular Docking Study

A molecular docking study was conducted on the isolated compound and the reference standard vitamin C, using the crystal structure of LOX‐3 in complex with protocatechuic acid (PDB ID: 1N8Q) [31]. This was downloaded from the Protein Data Bank. Kollman charges were added to the receptor and water molecules, and the co‐crystallized ligands were removed from the structure. The grid box was then fixed with dimensions of *x* = 40, *y* = 40, and *z* = 40, with the corresponding center coordinates of 30.708, −44.632, and −1.416 for *x*, *y*, and *z*, respectively. This screening was carried out using ViewerLite, AutoDock Vina, and Discovery Studio Visualizer software. Docking exhaustiveness was set to 8, and 10 poses were generated per ligand. The pose with the lowest binding energy and correct orientation was selected. Protocol validation was performed by re‐docking the co‐crystallized ligand protocatechuic acid, yielding an RMSD of 1.62 Å, confirming the reliability of the docking setup.

## Conflicts of Interest

The authors declare no conflicts of interest.

## Data Availability

The data that support the findings of this study are available in the Supporting Information of this article.
